# Incentive Preferences and Its Related Factors Among Primary Medical Staff in Anhui Province, China: A Cross-Sectional Study

**DOI:** 10.3389/fpubh.2021.778104

**Published:** 2022-01-05

**Authors:** Lingzhi Sang, Hongzhang Liu, Huosheng Yan, Jian Rong, Jing Cheng, Li Wang, Guoqiang Li, Yan Guo, Lei Zhang, Hong Ding, Guimei Chen, Ren Chen

**Affiliations:** ^1^School of Health Services Management, Anhui Medical University, Hefei, China; ^2^Affiliated Suzhou Hospital of Anhui Medical University, Suzhou, China

**Keywords:** primary medical staff, incentive preference, exploratory factor analysis, China's Anhui Province, human resource (HR)

## Abstract

**Background:** The shortage of primary medical staff is a major problem in the management of health human resources across many developing countries. By determining their preferences for various motivational and related factors, we examined the correlation between staff's motivation preference levels and staff turnover and turnover intention. This study aimed to further improve the incentive mechanism and to provide a reference for healthcare managers to formulate management strategies for the primary medical staff team.

**Methods:** A self-reported questionnaire survey was conducted to collect data. The basic survey content included demographic characteristics. The absolute level questionnaire and relative level questionnaire on the factors affecting motivation preference were used as the main assessment scales. A total of 1,112 primary health workers in Anhui Province were investigated. *T*-test, analysis of variance (ANOVA), exploratory factor analysis, and multiple linear regression analysis were performed to analyze the data.

**Results:** The survey respondents (45.1%) reported being satisfied with their relationship with colleagues, and other social relationships (46.9%). The Kaiser Meyer Olkin (KMO) value for the absolute preference degree for motivational factors was 0.951. Two factors (economic and non-economic factors), after using the maximum variance rotation axis method, explained 81.25% of the total variance. The regression analysis showed that primary medical staff members with low monthly income (*B* = −0.157) have a higher preference for non-economic factors; the higher the educational background (*B* = 0.133), the higher their preference for economic factors. In addition, with the increase in participants' age (*B* = −0.250), the preference for motivational factors gradually decreased.

**Conclusion:** Both economic and non-economic factors play an important role in enhancing the enthusiasm of primary medical workers and improving their work attitude. Managers should use their influence to stabilize the primary medical staff.

## Introduction

Primary medical staff are the basis for ensuring the smooth operation of primary-level medical institutions, and they shoulder the major responsibility of providing basic public health services. All the time, the role of this group has been continuously highlighted, and it is also reflected in a “World Health Report” by World Health Organization (WHO) (1). Since 2009, when China began to implement a series of new medical system reform measures, the concept of “strengthening primary health services” has been well-implemented. The improvement of primary medical services has always been the focus of China's health service reform.

The lack of healthcare personnel was brought to light at the beginning of 2020, when countries around the world were hit by the COVID-19 pandemic, and the disease prevention of the primary residents was not guaranteed. This is already becoming a global problem. The quality and density of health human resources are considered important reasons for the dearth of public health services (2, 3). The WHO emphasizes the chronic shortage of skilled health workers in health systems across countries in their report” (1). In response to this crisis, many low- and middle-income countries have widely used community health workers (CHW) to make up for the lack of basic public health services (4). In China, primary health services are generally provided by local primary medical institutions, which include community health service centers, township health centers, village clinics, and outpatient departments. For now, due to the poor working environment, insufficient salaries, and an inefficient incentive system, primary medical staff are not motivated to work. In addition to economic incentives, studies in other countries also suggest that the performance of primary medical staff is affected by retention policies (5–7). Some studies have also shown that basic training and continuing training and education for primary medical staff can improve their performance (8–10). However, research into primary medical staff in areas with a medium economic level, such as Anhui Province in China, is very limited.

Anhui is located in the east-central region of China with a moderate level of economic development. In recent years, Anhui Province has been gradually strengthening primary health care in rural areas (11). However, Anhui has a large population, and the distribution of health resources, especially that of human health resources, is still uneven. The latest statistics from the National Health Commission (12) show that in 2020, the total number of health workers in China is 12.9283 million, of which 842,300 are rural doctors and hygienists (only 6.52%, the lowest proportion in the past 10 years). The total number of health workers in Anhui Province is 503,000, of which 170,000 are primary health workers (only 33.8%). In terms of the distribution of medical institutions, the number of staff in community health service centers (stations) accounted for 14.5%, township hospitals accounted for 35.5%, village clinics accounted for 29.9%, and other primary medical institutions (including street hospitals and outpatient departments) accounted for 20.1%, there is still a big gap compared with the developed provinces in eastern China. Given the a forementioned problems, some Chinese scholars have proposed that health human resources need to be increased in communities and rural areas to ensure access to public health services for primary residents (13). Research based on Herzberg's two-factor theory also states that the key to improving staff enthusiasm is to invest in motivational factors, because to ensure high-quality medical staff, endogenous motivational factors are more desirable than exogenous motivational factors (14, 15).

However, there is also a lack of research on non-economic motivators (such as personal development and training, professional identity, etc.) in this province; Moreover, most of the policy suggestions given by previous studies are relatively shallow and outdated, unable to solve the new problems of the current period. Thus, our research aims to fill the above-mentioned gaps. It aimed to explore the current status of work motivation and its related factors among primary medical staff in Anhui Province, in order to stabilize the primary medical team and provide a basis to formulate efficient management strategies for the primary medical team. In turn, providing China's experience of health human resource management may guide relevant developing countries to formulate their local policy strategies.

## Methods

### Studying Setting

The economic level of Anhui is in the upper-middle level of the country. Compared with some provinces in western China, it is at a relatively high level, but compared with the eastern provinces and coastal areas, it is at a relatively low level. As of the end of 2020, the permanent population of Anhui Province was 71.19 million, of which the rural population was 46.52 million (65.3%), and the grassroots population accounted for a large proportion (12). The reasons for the selection of Anhui are as follows: (1) As a key area of China's primary healthcare reform, it has a very good policy environment; (2) Compared with other eastern provinces in China, there are relatively few studies on primary medical staff in Anhui Province; (3) Anhui has several primary medical personnel as shown above. In addition, the study was approved by the Ethical Committee of Anhui Medical University (AMUREC:20170260) and the study team obtained informed consent from all participants. Participants were made aware that they could withdraw at any time.

### Participants and Data Collection

The surveyed areas included North Anhui, Central Anhui, and Southern Anhui. Random sampling was used to select one county from each city in the three regions. A self-reported questionnaire survey was conducted among the primary medical staff at the selected counties and towns, and participation in the survey was anonymous and voluntary. The questionnaires in each region were collected by investigators with survey ability and experience and they immediately took back them after witnessing the subjects completed the questionnaire. Before conducting the survey, we provided several training sessions and exercises for the investigators. We also set the following inclusion criteria: (1) participants aged over 20 years; (2) work experience of at least 1 year; (3) the occupations of the medical staff were limited to doctors, nurses, pharmacists and administrative staff, etc. Before the study was officially initiated, a series of preliminary investigations were also conducted at the same location.

A total of 1,200 questionnaires were returned, of which 1,112 were valid questionnaires, and the effective response rate was 92.67% (1,112/1,200).

### Measures

After referring to related literature and expert consultation the study included three parts: (1) Participants' general demographic characteristics: gender (male, female), age, titles (primary, middle, or above), education (secondary school and below, college, bachelor's degree and above), working years (1–10, 11–20, 20 years, and above), marital status (including married, unmarried); (2) Work characteristics: monthly income (CNY 3000 and below, higher than CNY 3000), occupation (doctor, nurse, pharmacist, and administrative manager), work units (Mentioned above); (3) Area: the city and county (district) to which participants belong (according to the geographical characteristics of Anhui Province, the region was divided into northern Anhui, middle Anhui, and southern Anhui).

Based on previous studies and other survey tools related to work motivation (16–18), seven types of motivation factors, including income, working resource conditions, welfare, and career development prospects et al., were included in this study. Accordingly, we independently designed two sets of variables to determine participants' preferences for motivational factors. The first group of variables was ranked by participants' preference levels based on their satisfaction with these variables. The responses were ranked into five levels, ranging from 1 (“very dissatisfied”) to 5 (“very satisfied”). For the second group of variables, to measure the absolute level of participants' preferences for each motivational factor, a 5-point Likert scale was used to assess the motivational factors that increase participants' effort. It should be noted that the motivational factors in this scale are divided into 11 categories. For each type of motivational factor, the following question was asked: “If the salary level (reward method/benefits/...) improves, how much will your work effort increase?”. The responses were made on a scale ranging from 1 (“no improvement”) to 5 (“considerable improvement”). In addition, basic information on participants was obtained: gender, age, educational background, working years, marital status, average monthly income, job position, and type of primary medical institution. As quality control personnel, undergraduates and graduate students majoring in preventive medicine and health management explained the questionnaires at the survey site, and collected and checked the questionnaires. It is worth mentioning that all research assistants were trained in data collection.

### Statistical Analyses

We used SPSS 26.0 for data analysis, and the mean and composition ratio were used to describe participants' degree of preferences for various motivational factors. *T*-test and analysis of variance (ANOVA) were used to perform a single-factor analysis of the incentives of binary variables (gender, marital status, monthly income). Then, we divided the age of primary medical staff into four groups: ≤ 35, 36–45, and >45 years; thus, the age of primary medical staff was transformed from a continuous variable to a categorical variable. One-way ANOVA was performed for multiple categorical variables (including age, education, institution, occupation, and region). If there was no homogeneity of variance, the Kruskal-Wallis *H*-test was performed. We also constructed a multiple linear regression model to analyze the differences in the degree of participants' preference for motivation factors under different conditions (α = 0.05).

The exploratory factor analysis method was used to analyze the inner level of the participants' demand for motivating factors, mainly to extract the latent factors among the included variables. First, we measured the KMO value of the selected scale and checked whether the *p* <0.001 using the Bartlett sphere method, which then determined whether this set of variables is suitable for factor analysis. Second, we use the principal component method to extract two factors, which would explain the variance in all variables. The maximum variance rotation axis method was used to orthogonally rotate the factor load matrix, to obtain the rotated factor load matrix. Finally, variables with load values> 0.5 were attributed to a common factor, and they were labeled “non-economic factors” and “economic factors.” The reliability of the scale is Cronbach's α coefficient of 0.89, and the results of confirmatory factor analysis showed the scale has good reliability.

## Results

### Socio-Demographic Characteristics of Participants

A total of 1,112 participants were studied, of whom 609 were women and 503 were men, accounting for 54.8 % and 45.2 % of the participants, respectively. The participants were 36–45 years old, with an average age of (40.22 ± 8.453). There were 482 people with a high school education or less, accounting for 43.3% of the total, and 545 medical staff with a monthly income of CNY 3000 and below, accounting for 49.0% of the total. Among different occupations, there were 579 doctors (52.1%) and 235 nurses (21.1%). (See Table 1).

**Table 1:** Socio-demographic characteristics of participants (*n* = 1,112).

**Demographic variable**	**Composition ratio (%)**	***t/F***	***P*-value**
Sum	1,112 (100)	–	–
Gender		1.503	0.199
Male	503 (45.2)		
Female	609 (54.8)		
Age (years)		2.43	<0.001^**^
<36	296 (26.6)		
36 45	502 (45.1)		
>45	314 (28.2)		
Education level		6.508	<0.001^**^
secondary school and below	482 (43.3)		
Associate degree	435 (39.1)		
Bachelor degree and above	195 (17.5)		
Marital status		5.71	0.017^*^
Married	1,017 (91.5)		
Unmarried	95 (8.5)		
Monthly income (CNY)		3.45	<0.001^**^
CNY 3000 and below	545 (49.0)		
higher than CNY 3000	567 (51.0)		
Occupation		2.973	0.019^*^
Physicians	579 (52.1)		
Pharmacists	235 (21.1)		
Nurses	298 (26.8)		
Regions		0.745	0.561
North of Anhui	770 (69.2)		
Middle of Anhui	209 (18.8)		
South of Anhui	133 (12.0)		
Work units		1.370	0.242
CHC	171 (15.4)		
CHS	688 (61.9)		
Township hospitals	171 (15.4)		
Village clinics	82 (7.4)		

*CHC, community health service center; CHS, community health service station*.

*Bold values:*

* *P <0.05,*

** *P <0.001*.

### Description of the Relative Level of Motivation Factor Preference and Satisfaction

Primary medical staff members were found to be highly satisfied with the three motivational factors of colleague relations, social relations, and management styles, but satisfaction with income, welfare, and sense of accomplishment is relatively low. 532 people (47.8%) and 522 people (46.9%), respectively reported generally satisfaction with their career achievements and social relationships, and nearly 95% of them felt that their relationships with colleagues were at different levels of satisfaction. Both 13 people (1.2%) were very dissatisfied with their social and colleague relationships. The preference of primary medical staff in Anhui Province for economic factors was determined to be higher than that for non-economic factors; this result was obtained from the average results of various incentive factors. (See Table 2).

**Table 2:** Description of the relative level of incentive factors preference and satisfaction degrees.

**Incentive types**	**Incentive factors**	**M ± SD**	**Composition ratio [n (%)]**				
			**Very dissatisfied**	**Not so satisfied**	**Generally satisfied**	**Relatively satisfied**	**Very satisfied**
Economic incentives	Income	2.48 ± 0.97	286 (25.7)	387 (34.8)	287 (25.8)	127 (11.4)	25 (2.2)
	Welfare	2.54 ± 1.07	326 (29.3)	291 (26.2)	295 (26.5)	164 (14.7)	36 (3.2)
	Working resource	2.90 ± 0.97	89 (8.0)	262 (23.6)	480 (43.2)	228 (20.5)	53 (4.8)
	Management system	3.17 ± 0.97	55 (4.9)	186 (16.7)	483 (43.4)	294 (26.4)	94 (8.5)
	Environment	2.24 ± 0.95	50 (4.5)	153 (13.8)	495 (44.5)	313 (28.1)	101 (9.1)
Non-economic incentives	Training opportunities	2.84 ± 1.03	119 (10.7)	272 (24.5)	460 (41.4)	193 (17.4)	68 (6.1)
	Promotion of title	2.95 ± 0.98	85 (7.6)	245 (22.0)	493 (44.3)	224 (20.1)	65 (5.8)
	Pride and accomplishment	2.05 ± 0.97	73 (6.6)	194 (17.4)	532 (47.8)	232 (20.9)	81 (7.3)
	Colleague relations	3.78 ± 0.84	13 (1.2)	43 (3.9)	335 (30.1)	502 (45.1)	219 (19.7)
	Social relationship	3.46 ± 0.82	13 (1.2)	78 (7.0)	522 (46.9)	380 (34.2)	119 (10.7)

### Exploratory Factor Analysis on the Levels of Various Motivational Factors

We used exploratory factor analysis to extract the potential factors from the preference variables of motivational factors; through the interpretation of the meaning of these factors, the level of motivational factor needs of primary medical staff were found. The KMO value of the measure of absolute preference degree of motivational factors was 0.951, and the result of Bartlett's sphere test was statistically significant at *P* < 0.001, indicating that this set of variables is suitable for further factor analysis.

First, two value factors were extracted by the principal component method, and then the axis of maximum variance method was used. The rotated factor loading matrix was obtained after its orthogonal rotation. The test results showed that two factors can explain 81.25% of the variance of all variables. Second, we attributed variables with load values > 0.5 to a common factor, and then named them as non-economic factor and economic factor preferences based on the content reflected by the explanatory variables of each factor. (See Table 3).

**Table 3:** Exploratory factor analysis on the levels of various motivational factors.

**Factors**	**Factor extraction**	
	**Economic**	**Non-economic**
	**factors (F1)**	**factors (F2)**
Salary level	0.857*	0.336
Reward method	0.841*	0.387
Welfare	0.829*	0.416
Guarantee mechanism	0.809*	0.433
Working environment	0.498	0.728*
Medical equipment	0.426	0.749*
Management method	0.386	0.797*
Colleague relations	0.318	0.828*
Sense of pride and accomplishment	0.383	0.804*
Social relationship	0.343	0.845*
Cumulative variance contribution rate (%)	81.251	43.962
Internal consistency coefficient	0.940	0.945
Means of each motivation factor	3.623	3.574

Bold values: *Factor loadings ≥ 0.5.

### Multiple Linear Regression Analysis on the Preference Degree of Various Motivational Factors and Related Factors

We took non-economic factors and economic factors as dependent variables, and included statistically significant factors as independent variables in a single factor analysis. A multiple linear regression analysis was performed, and the results showed that the monthly income (*B* = −0.157, *P* < 0.001) of primary medical staff was negatively correlated with the preference degree of non-economic factors. Similarly, age (*B* = −0.250, *P* = 0.020) and the preference degree of economic factors were also negatively correlated. However, education (*B* = 0.133, *P* < 0.001) were positively correlated with the degree of preference for economic factors. (See Table 4).

**Table 4:** Multiple linear regression analysis on the preference degree of various motivation factors and related factors.

**Items**		**Unstandardized coefficients**		**Standardized coefficients**	***P*-value**	**95% CI**
		***B***	**SE**	**β**		
Economic factors	Constant term	11.170	0.832		<0.001**	(9.539, 12.802)
	Age (years)	−0.250	0.148	−0.054	0.020*	(−0.542, 0.041)
	Education level	0.133	0.164	0.025	<0.001**	(−0.188, 0.455)
	Marital status	0.083	0.433	0.006	0.848	(−0.766, 0.932)
	Monthly income (CNY)	1.667	0.231	0.211	0.092	(1.215, 2.119)
	Occupation	0.459	0.094	0.144	0.416	(0.275, 0.644)
Non–economic factors	Constant term	14.208	0.745	14.208	<0.001**	(12.745, 15.671)
	Age (years)	0.878	0.207	0.126	0.239	(−0.418, 0.104)
	Education level	0.063	0.147	0.013	0.670	(−0.226, 0.351)
	Marital status	0.120	0.388	0.010	0.758	(−0.641, 0.881)
	Monthly income (CNY)	−0.157	0.133	−0.039	<0.001**	(0.473, 1.284)
	Occupation	0.343	0.084	0.122	0.289	(0.178,0.509)

*P < 0.05, **P < 0.001.

## Discussion

Our study found that both economic incentives and non-economic incentives may have direct or indirect impacts on primary medical staff in Anhui Province, and we also found some related factors (such as monthly income, age, and educational background) from the data analysis, and they may also play a potential role. The following is the discussion from these three aspects, and based on this, to improve the job satisfaction of primary medical staff, enhance their work enthusiasm and self-confidence.

### Research on Economic Incentives

The results of this study showed that at present, welfare and income are the two motivational factors with which primary medical staff in Anhui are the most dissatisfied. This also shows that their expectations for material rewards are often high, which fits the results of the exploratory factor analysis. In other words, when comparing averages of the various incentive factors, the preference of basic-level medical staff for economic factors is higher than that for non-economic factors. This result is consistent with the results of a study in China (17), in which primary medical staff was found to value material incentives over non-material incentives. At the same time, a cross-sectional survey on the job satisfaction of primary medical staff in Shandong Province, China (18) also pointed out that the most dissatisfactory aspect of primary-level health workers is their salary. In addition, two Chinese studies (19, 20) pointed out that health workers have a strong demand for higher wages and benefits. This situation is more common in primary medical institutions, and has been a long-standing problem. However, a study in Lithuania (21) showed that most primary doctors accepted their current working conditions, and correspondingly, their job satisfaction was high. The reason for this difference could be the different healthcare conditions between the two countries: Lithuania is in southern Europe with a small population and a small area, so it is convenient for the distribution of health human resources, especially when it comes to the distribution of these resources in communities and villages. Moreover, the country has a high degree of medical specialization; hence, it is convenient to carry out medical training for general practitioners. However, China has a large area with a large population, and the unfair distribution of health resources among regions is inevitable. Faced with limited health resources, it is difficult for medical services to be easily accessible to communities and in rural areas.

A previous job satisfaction survey of general practitioners in Hubei Province found that young doctors may leave their jobs because of low job satisfaction (22), which fits well with the conclusion of this study, i.e., age and material incentive preference is negatively correlated. It is reasonable that young doctors tend to value material incentives more than older doctors. As young people, they are inferior to older doctors in wealth and social status, which encourages these young people to work harder, such that the level of their salary often becomes an important indicator of their progress.

As for Anhui Province, the reasons for the low satisfaction of primary medical staff may be many. First, due to limited health human resources, the workload of primary medical staff is often larger than that of other medical staff, so overtime is more common. Second, the subsidy systems of medical institutions are imperfect, and overtime subsidies cannot be paid on time, such that the input and return of the workload of the staff ends up being disproportionate (23). The basic public health subsidies for rural doctors are in place (24), which leads to the dissatisfaction of basic-level medical staff with remuneration.

Therefore, it is particularly important and even urgent to establish a scientific and reasonable salary and incentive system. China has begun to implement a reform of the medical insurance payment method, and the “pay by individual” method has begun to take effect. This payment method can transform the increased investment in health into an improvement in the income and welfare of medical staff (25). At the same time, it not only pays attention to the salary of workers, but also pays attention to the fairness and reasonableness of the distribution process (26). China's health department should consider giving this system greater operational and personnel autonomy to stimulate its innovative vitality (27, 28).

### Research on Non-economic Incentives

The results of this study found that primary medical staff in Anhui Province valued economic factors more than non-economic factors, but the difference between the two was not big, which shows that non-economic incentives also have a positive effect on workers. Previous studies have shown that the incentive effects of non-economic factors include the improvement of the work environment and an enhanced sense of work accomplishment (29). A survey of rural doctors in China showed that the economic returns of primary care in primary areas are much lower than that of high-level hospitals in cities (30). The core of the problem faced by primary medical staff is salary inequality; this is because the generation of income depends largely on higher service fees and paper publishing income (31). A satisfaction survey of community health workers also showed that the salary of primary medical staff is relatively lower than that of medical staff in higher-level hospitals in cities, and it is difficult for these primary health workers to get a big increase in their income in a short period of time. In summary, the non-economic incentives of the primary medical staff need to be strengthened. Some scholars believe that good colleague relations and the support of superior leaders will bring job satisfaction (32). A research report on health workers in dozens of low and middle-income countries around the world suggested that fair treatment and mutual respect among colleagues, supervisors, organizations, and patients will also affect intrinsic motivation and enthusiasm (33). Anhui Province is an area with a medium economic level and a large proportion of its population in primary areas. In terms of the management strategy of primary health workers, good non-economic incentives have an important impact on improving their motivation to work, which undoubtedly fits the current situation in Anhui Province.

In this regard, we recommend that health departments take active and effective action to provide non-economic incentives to primary medical staff, such as improving their working environment. A study in Ghana, which is a developing country, found that poor working environment and limited job prospects are the prominent negative factors experienced by primary health workers (34). Systematic research on the motivation and retention of health workers in developing countries by the scholars of the World Health Organization determined that adequate supply and proper infrastructure are two factors that can significantly improve morale and increase employee motivation (35). It is time to bring in these experiences and apply them to some developing regions in China.

### Analysis of the Reasons for the Influence of Different Motivational Factors

It is worth mentioning that the multi-factor analysis of the level of incentive preference shows that primary medical staff members with low monthly income have a higher preference for non-economic factors. We speculate that incentives relating to non-economic factors may be at work in increasing the workers' sense of accomplishment about their work, such as, in the case of an improvement in social relations at the workplace. Health workers with higher educational backgrounds have a higher preference for economic factors. It may be that they have received higher medical education and training, and their corresponding training costs were higher than those of other health workers. Therefore, they place higher expectations on salary and benefits. However, once their actual economic income differs too much from the expected value, they may tend to feel that their effort and income cannot match (36), which arouses their dissatisfaction. In addition, the differences in incentive preferences between medical staff due to differences in their ages are not consistent with the results of a study of community general practitioners (16); the reason may be that the subjects of our study were rural health workers. Compared with general practitioners, the working environment of rural health workers is worse, and capital investment and operation are more difficult.

## Limitations

Although we conducted a lot of quality control before the field survey and data analysis, we must admit that this study still has some limitations. First, the sample is only part of the primary medical staff in Anhui Province; hence, their overall situation may not be able to describe all Chinese health staff. the sample mainly includes participants from Anhui Province, but does not include other parts of China. Therefore, we did not conduct a comparative analysis. Finally, some confounding factors are inevitable, such as some traditional ideas in rural areas.

## Conclusions

This study investigated the incentive preference level of primary health workers in Anhui Province, China, and we also found some related factors that affect their incentive preference. The results show that the monthly income, education, and age of health workers may affect their incentive preferences. For young people with a low monthly income and high educational level, sufficient attention should be paid to their current life and work status. This was the key population of this study. In addition, managers should combine economic incentives with non-economic incentives; adapt measures to local conditions and individual preferences, which may vary; refer to the experience of domestic and foreign research to improve the welfare system of primary medical staff and increase their job satisfaction; and gradually increase their income. At the same time, enhancing the enthusiasm and self-confidence of the staff in their current work and creating an optimistic working atmosphere must also be looked into.

## Data Availability Statement

The original contributions presented in the study are included in the article/supplementary material, further inquiries can be directed to the corresponding author/s.

## Ethics Statement

The studies involving human participants were reviewed and approved by the Ethical Committee of Anhui Medical University. The patients/participants provided their written informed consent to participate in this study.

## Author Contributions

LS and HL participated in the survey, the data analysis, and writing of the article. HY, JR, GL, YG, and LZ contributed to the data collection and screening. JC, LW, and HD were involved in the data analysis and participated in the literature research. GC and RC participated in the design of the study, contributed to quality control and data processing, and revised the manuscript. All authors have read and approved the final version.

## Funding

This work was supported by the National Nature Science Foundation of China (grant numbers: 71874002, 72174001) and Anhui Natural Science Foundation (No. 1808085QH252). The sponsors and funding agencies had no role in design and implementation of this study or the preparation of this article.

## Conflict of Interest

The authors declare that the research was conducted in the absence of any commercial or financial relationships that could be construed as a potential conflict of interest.

## Publisher's Note

All claims expressed in this article are solely those of the authors and do not necessarily represent those of their affiliated organizations, or those of the publisher, the editors and the reviewers. Any product that may be evaluated in this article, or claim that may be made by its manufacturer, is not guaranteed or endorsed by the publisher.

## Abbreviations

ANOVA: analysis of variance
CHW: community health workers
SD: Standard Deviation
M: means.
